# Effect of prediabetes on the long-term all-cause mortality of patients undergoing percutaneous coronary intervention: A protocol for systematic review and meta analysis

**DOI:** 10.1097/MD.0000000000021623

**Published:** 2020-08-14

**Authors:** Rui Shi, Kai-yue Diao, Ke Shi, Yue Gao, Shan Huang, Ying-kun Guo, Zhi-gang Yang

**Affiliations:** a Department of Radiology, West China Hospital, Sichuan University, Chengdu, Sichuan; b Department of Radiology, Key Laboratory of Birth Defects and Related Diseases of Women and Children of Ministry of Education, West China Second University Hospital, Sichuan University, China.

**Keywords:** metabolic abnormality, percutaneous coronary intervention, prediabetes

## Abstract

**Background:**

Prediabetes is an abnormal metabolic state that develops prior to the onset of diabetes with proven to common comorbid states of coronary artery disease. However, whether prediabetes worsens prognosis after percutaneous coronary intervention remains controversial. The aim of this study is to summarize previous cohort studies and to specify the impact of prediabetes on the long-term outcomes after percutaneous coronary intervention.

**Methods:**

This meta-analysis will be performed according to the Preferred Reporting Items for Systematic Review and Meta-Analysis guidelines for conducting and reporting meta-analysis data. Pubmed, Embase and Google scholar will be systematically searched, and supplemented with manual searches of the included reference lists to identify cohort studies. Pooled effects on the discontinuous variables will be expressed by adjusted hazard ratios with 95% confidence intervals. All analyses will be performed with Stata 15.0 (StataCorp LP, College Station, TX).

**Results:**

The results of this study will be published in a peer-reviewed journal.

**Conclusion:**

This systematic review will provide new information and help enhance clinical decision-making on management of these patients.

**Registration number:**

INPLASY202060079.

## 1 Introduction

Prior to the progression to diabetes, some patients experience long-term glycometabolic abnormalities, which is an intermediate status with borderline high plasma glucose levels, referred to as prediabetes.^[1,2]^ Prediabetes, including impaired glucose tolerance (IGT), impaired fasting glucose (IFG-WHO: 6.1-6.9 mmol/L or IFG-ADA: 5.6–6.9 mmol/L) and elevated HbA1c (HbA1c-ADA: 39–47 mmol/mol or HbA1c-NICE: 42–47 mmol/mol),^[3,4]^ is also 1 of the most common comorbid states of coronary artery disease (CAD).^[5,6]^ Diabetes leads to a deterioration in coronary plaque burden and an increased event risk after stenting.^[7–9]^ The association between the risk of cardiovascular disease and the loss of glucose control have been thoroughly investigated in the general population, revealing prediabetes as a risk factor for cardio-cerebrovascular events and all-cause mortality.^[10,11]^

The majority of prediabetes patients develop diabetes, although lifestyle changes can prevent this progression.^[12,13]^ Recent studies have focused on the long-term clinical prognosis of prediabetes patients after percutaneous coronary intervention (PCI). These studies led to inconsistent conclusions mainly due to differences in endpoint assessments and the relatively small number of enrolled patients. Whether prediabetes worsens the long-term prognosis of patients who underwent PCI is now largely debatable.

In view of these inconsistencies, we performed a meta-analysis of cohort studies to assess the association between prediabetes and long-term outcomes, including all-cause death, myocardial infraction (MI) and major adverse cardiovascular events (MACE) after PCI.

## 2 Methods and analysis

This meta-analysis will be performed according to the Preferred Reporting Items for Systematic Review and Meta-Analysis (PRISMA) guidelines for conducting and reporting meta-analysis data.^[14]^ The protocol for this review has been registered on the INPLASY website (https://inplasy.com/inplasy-2020-6-0079/) and the study registration number is INPLASY202060079. If adjustments are needed throughout the study, we will update the details in the final version.

### 2.1 Inclusion and exclusion criteria

We propose to inclusion prospective/retrospective cohort studies provide long-term events (>1 year), including all-cause mortality, MI or MACE in PCI-treated CAD patients. All the research objects in these studies were completed PCI (no matter selective or primary) in hospitalization. Studies with prediabetes defined as IFG (IFG-WHO: 6.1–6.9 mmol/L or IFG-ADA: 5.6–6.9 mmol/L), IGT (2 h plasma glucose 7.8–11.0 mmol/L during an oral glucose tolerance test), or raised HbA1c (HbA1c-ADA: 39–47 mmol/mol or HbA1c-NICE: 42–47 mmol/mol) will be eligible for inclusion. Researches will be excluded if adverse long-term outcomes are not amongst the clinical endpoints and those meta-analyses, reviews, letter to editors or case reports. No language or date restrictions will be applied. In addition, studies defined prediabetes in combined status (eg, combined IFG with raised HbA1c) will not be include in our final analysis for these patients possess different physiopathological mechanisms and are more likely to progress to diabetes.

### 2.2 Search strategy

Embase, Pubmed, and Google scholar will be comprehensively searched up to the 1st September 2019, and supplemented with manual searches of the included reference lists to identify cohort studies. Search strategies will be developed and reviewed by 2 experienced librarian investigators. Any discrepancy is resolved by consensus. An example of search string for Pubmed is shown as follow:

#1 (((((“Blood Glucose”[Mesh]) OR “Glucose Intolerance”[Mesh]) OR “Prediabetic State”[Mesh]) OR “Hyperglycemia”[Mesh]) OR “Hemoglobin A, Glycosylated”[Mesh])

#2 (((((((((((“Prediabetic State”[Text Word]) OR Hyperglycemia[Text Word]) OR “impaired fasting glucose”[Text Word]) OR “impaired glucose tolerance”[Text Word]) OR “pre-diabetes”[Text Word]) OR prediabetes[Text Word]) OR “borderline diabetes”[Text Word]) OR “higher risk of diabetes ”[Text Word]) OR “ high risk of diabetes”[Text Word]) OR hemoglobin A1c [Text Word]) OR HbA1c[Text Word])

#3 #1 OR #2

#4 ((((“Cardiovascular Diseases”[Mesh]) OR “Mortality”[Mesh]) OR “Death”[Mesh]) OR “Fatal Outcome”[Mesh])

#5 (((((((((“ischemic heart disease”[Text Word]) OR “myocardial infarction”[Text Word]) OR “cardiovascular events”[Text Word]) OR “cerebrovascular disease”[Text Word]) OR “stroke”[Text Word]) OR “cerebral infarction”[Text Word]) OR Mortality[Text Word]) OR fatal[Text Word]) OR death[Text Word])

#6 #4 OR #5

#7 (((“Coronary Diseases”[Mesh]) OR “Coronary Artery Disease”[Mesh]) OR “Heart Disease”[Mesh])

#8 ((((((PCI [Text Word]) OR revascularization [Text Word]) OR invasively [Text Word]) OR intervention [Text Word]) OR angioplasty [Text Word]) OR “percutaneous coronary intervention”[Text Word])

#9 #7 OR #8

#10 #3 AND #6 AND #9

#11 animals [MeSH Terms]

#12 humans [MeSH Terms]

#13 #11 NOT #12

#14 #10 NOT #13

#15 (“Risk”[Mesh]) OR risk [Text Word]

#16 #14 AND #15

#17 (((((((((((epidemiologic studies[MeSH Terms]) OR cohort studies[MeSH Terms]) OR “epidemiologic”[Text Word]) OR “cohort”[Text Word]) OR “longitudinal”[Text Word]) OR “follow up”[Text Word])) OR observational[Text Word]) OR prospective[Text Word])

#18 #16 AND #17

### 2.3 Study selection

Studies searched using above search strategy will be imported into Endnote version X9 software to manage and reviewed by 2 investigators independently. Any discrepancies between the reviewers are resolved by consensus through discussions. Papers from the same research team will be compared to minimize data duplication. When similar studies derived from the same cohort are found, the most recent publications will be included. Relevant studies chosen by at least 1 author will be retrieved. For documents that be potential to meet the inclusion criteria after titles and abstracts screening, full papers will be retrieved. A Flow of studies retrieving will be developed using the PRISMA guidelines.

### 2.4 Data extraction

Standardized extraction form will be made by an experienced researcher with 4-year of experience performing systematic reviews and meta-analysis (K.Y. Diao) using Microsoft Excel. The following information will be extracted from each of the included studies by another 2 investigators (R. Shi and G. Yue): study design, first author, year of publication, definition of prediabetes, follow-up duration, patient characteristics (acute coronary syndrome (ASC), stable CAD or both), sample size, outcomes (including all-cause mortality, MI or MACE), test events, control events and adjusted Hazard Ratios (HRs) with 95% confidence intervals (CIs) for prediabetes. If the adjusted HRs are not reported. We will attempt to contact authors and request for additional information when the information is inadequate. Otherwise, the adjusted RRs with 95% CIs will be recorded. Lost information will be conversed or calculated from the available data if possible. Sensitivity or subgroup analysis will be performed to according to data accuracy (directly extracted or calculated) to avoid the heterogeneity caused by inaccurate information as much as possible.

### 2.5 Quality evaluation

Quality assessment of the included studies will be performed using the Newcastle-Ottawa quality assessment scale (NOS).^[15]^ NOS, assessing 3 parameters (selection, comparability, and exposure assessment) of study quality, is a validated quality assessment instrument for nonrandomized trials. The maximum total score of 9 for NOS (maximum score of 4 for selection, 2 for comparability, and 3 for exposure). We grade the quality of the studies as high (>=5 stars) or low (<5 stars) according to the NOS standard in our meta-analysis, and this will serve as 1 of the bases for the subgroups. Two investigators (R. Shi and Y. Gao) assess the study quality independently. Disagreements are resolved by discussion between the reviewers, with a third author as a final arbiter (K.Y. Diao) with 4-years of experience performing systematic reviews and meta-analysis.

### 2.6 Statistical analysis

#### 2.6.1 Data synthesis

Statistical analyses are performed with Stata Version 15.0 (StataCorpLP, College Station, TX). Adjusted HRs with 95% CIs for long-term adverse events, including all-cause mortality, MI or MACE in PCI-treated CAD patients, are extracted and polled. We included studies defined prediabetes with combined status as a separate category for analysis to explore whether these patients are at higher risk of adverse prognosis than those with isolated IFG, IGT or elevated HbA1c. If there are outcomes could not be included in this meta-analysis as plan or reported only once, the results will be presented separately or in discussion part.

Statistical heterogeneity is assessed using the *Q*-statistic (significant at PQ<0.1). The extent of heterogeneity is assessed using the inconsistency index (*I*^2^) (0–40%, absent or mild heterogeneity; 30% to 60%, moderate heterogeneity; 50% to 90%, substantial heterogeneity; and 75% to 100%, considerable heterogeneity). Clinical and methodological differences between the included studies will be considered. We use a random effects model over the fixed effects model, although no significant heterogeneity is indicated.

#### 2.6.2 Subgroup analysis

The diagnostic criteria of prediabetes are the strongest confounder of long-term outcomes after PCI, and considered as a potential source of heterogeneity. We plan to perform a further subgroup analyses of the outcomes according to the prediabetes diagnosis (IFG, IGT or HbAc1) as well as ethnicity, the possibility of enrolling patients with diabetes, and population type (acute coronary syndrome, stable angina or both). χ2 will be used to test for subgroup differences.

#### 2.6.3 Sensitivity analysis

Sensitivity analysis will be performed by recalculating the pooled relative risk following the omission of single studies in a step-wise manner. We will also perform sensitivity analysis by excluding studies without adequate information.

#### 2.6.4 Publication bias

Publication bias will be assessed quantitatively for the outcomes with more than 10 studies. Begg funnel plots and Egger test will be used. Publication bias will be considered when an asymmetrical funnel plot or a *P*-value of <.10 on Egger test is detected. Trim and fill method will be used to estimate how many studies are in the asymmetric part and then trim off the asymmetric outlying part of the funnel. The final estimate of the true mean and its variance are based on the filled funnel plot.

### 2.7 Evidence quality evaluation

We will evaluate the evidence grade according to guidelines of the Grading of Recommendations, Assessment, Development, and Evaluation (GRADE) system.^[16]^ Sequential assessment of the evidence quality will be used, following by an assessment of the risk–benefit balance. A total of 4 levels classified by the GRADE for the quality of evidence: very low quality, low quality, moderate quality, and high quality.

## 3 Discussion

We design this protocol for a meta-analysis to analyzed the association between prediabetes and long-term adverse outcomes after PCI. In addition, we further assess the impact of different definition of prediabetes on the outcomes.

There is evidence to suggest that hyperglycemia is an accurate marker of increased all-cause mortality in the general population.^[11]^ Increasing numbers of patients with established CAD show concomitant prediabetes.^[17]^ Whether prediabetes affects the long-term prognosis after PCI is not yet clear. Although many studies have paid attention to this issue, the results are inconsistent, and it is necessary to conduct a meta-analysis on this controversial issue. We have reason to believe that our systematic review will provide new information and help enhance clinical decision-making on management of these patients.

## Author contributions

SR, DKY, GYK, and YZG conceived this study. SR and DKY developed the study protocol and will implement the systematic review under the supervision of GYK and YZG. SK and GY will provide the statistical analysis plan of the study and will conduct data analysis. SK, GY and SR will perform the study search, screening, and extraction of data whereas YZG and DKY will review the work. SR wrote the first manuscript draft and all authors gave input to the final draft of the protocol. YZG is guarantor of the review.

## References

[1] Tabak AG Herder C Rathmann W Prediabetes: a high-risk state for diabetes development. Lancet 2012;379:2279–90. 22683128 10.1016/S0140-6736(12)60283-9 PMC3891203

[2] Echouffo-Tcheugui JB Niiranen TJ McCabe EL Lifetime prevalence and prognosis of prediabetes without progression to diabetes. Diabetes Care 2018;41:e117–8. 29724784 10.2337/dc18-0524 PMC6014534

[3] World Health Organization (WHO) Consultation. Definition and diagnosis of diabetes and intermediate hyperglycaemia. 2006. Available at: http://www.who.int/diabetes/publications/Definition%20and%20diagnosis%20of%20diabetes_new.pdf.

[4] Report of the expert committee on the diagnosis and classification of diabetes mellitus. Diabetes Care 2003;26: Suppl 1: S5–20. 12502614 10.2337/diacare.26.2007.s5

[5] Kiviniemi AM Lepojarvi ES Tulppo MP Prediabetes and risk for cardiac death among patients with coronary artery disease: the artemis study. Diabetes Care 2019;42:1319–25. 31076416 10.2337/dc18-2549

[6] Fujihara K Igarashi R Yamamoto M Impact of glucose tolerance status on the development of coronary artery disease among working-age men. Diabetes Metab 2017;43:261–4. 27712966 10.1016/j.diabet.2016.09.001

[7] Shi R Shi K Yang ZG Serial coronary computed tomography angiography-verified coronary plaque progression: comparison of stented patients with or without diabetes. Cardiovasc Diabetol 2019;18:123. 31551077 10.1186/s12933-019-0924-z PMC6760061

[8] Venuraju SM Lahiri A Duration of type 2 diabetes mellitus and systolic blood pressure as determinants of severity of coronary stenosis and adverse events in an asymptomatic diabetic population: PROCEED study. Cardiovasc Diabetol 2019;18:51. 31014330 10.1186/s12933-019-0855-8 PMC6480794

[9] Wang Q Liu H Ding J Cardiac versus non-cardiac related mortality following percutaneous coronary intervention in patients with insulin-treated type 2 diabetes mellitus: a meta-analysis. Diabetes Ther 2018;9:1335–45. 29779197 10.1007/s13300-018-0444-y PMC5984945

[10] Huang Y Cai X Chen P Associations of prediabetes with all-cause and cardiovascular mortality: a meta-analysis. Ann Med 2014;46:684–92. 25230915 10.3109/07853890.2014.955051

[11] Huang Y Cai X Mai W Association between prediabetes and risk of cardiovascular disease and all cause mortality: systematic review and meta-analysis. BMJ 2016;355:i5953. 27881363 10.1136/bmj.i5953 PMC5121106

[12] Van Name MA Camp AW Magenheimer EA Effective translation of an intensive lifestyle intervention for hispanic women with prediabetes in a community health center setting. Diabetes Care 2016;39:525–31. 26908915 10.2337/dc15-1899 PMC4806769

[13] Armato JP DeFronzo RA Abdul-Ghani M Successful treatment of prediabetes in clinical practice using physiological assessment (STOP DIABETES). Lancet Diabetes Endocrinol 2018;6:781–9. 30224284 10.1016/S2213-8587(18)30234-1

[14] Moher D Liberati A Tetzlaff J Preferred reporting items for systematic reviews and meta-analyses: the PRISMA statement. PLoS Med 2009;6:e1000097. 19621072 10.1371/journal.pmed.1000097 PMC2707599

[15] The Newcastle-Ottawa Scale (NOS) for assessing the quality of nonrandomised studies in meta-analyses. Available at: http://www.ohri.ca/programs/clinical_epidemiology/oxford.asp.

[16] Atkins D Best D Briss PA Grading quality of evidence and strength of recommendations. BMJ 2004;328:1490. 15205295 10.1136/bmj.328.7454.1490 PMC428525

[17] Standl E Khunti K Hansen TB The global epidemics of diabetes in the 21st century: current situation and perspectives. Eur J Prev Cardiol 2019;26: 2_suppl: 7–14. 10.1177/2047487319881021 31766915

